# Long-term risk for end-stage kidney disease and death in a large population-based cohort

**DOI:** 10.1038/s41598-018-26087-z

**Published:** 2018-05-16

**Authors:** Emanuel Zitt, Constanze Pscheidt, Hans Concin, Reinhard Kramar, Raphael S. Peter, Jan Beyersmann, Karl Lhotta, Gabriele Nagel

**Affiliations:** 1 Department of Nephrology and Dialysis, Academic Teaching Hospital Feldkirch, Feldkirch, Austria; 2 Vorarlberg Institute for Vascular Investigation and Treatment (VIVIT), Academic Teaching Hospital Feldkirch, Feldkirch, Austria; 3 Agency for Preventive and Social Medicine, Bregenz, Austria; 4 Institute of Epidemiology and Medical Biometry, Ulm University, Ulm, Germany; 5 Austrian Dialysis and Transplant Registry, Rohr im Kremstal, Austria; 6 Department of Statistics, Ulm University, Ulm, Germany

## Abstract

Knowledge of metabolic risk factors for end-stage kidney disease (ESKD) in the general population is limited when considering the competing event death in risk analysis. The aim of our prospective observational study was to investigate how blood pressure and metabolic factors might influence the risks for ESKD and death before ESKD in a large Austrian population-based cohort with long-term follow-up. 177,255 participants (53.8% women; mean age 42.5 years) were recruited between 1988 and 2005 and linked to the Austrian Dialysis and Transplant Registry and the National Mortality Registry. Over a mean follow-up of 16 years 358 participants reached ESKD and 19,512 participants died. Applying fully adjusted cause-specific Cox proportional hazards models elevated fasting blood glucose, hypertension, hypertrigylceridemia and hypercholesterolemia were associated with a higher relative risk for ESKD than for death before ESKD, whereas elevated γ-glutamyltransferase was associated with an increased relative risk of death but not ESKD. Results were similar using continuous or categorical exposure variable measures in the general cohort but differed in selected high-risk populations. These findings might help improve the design of renal risk factor modification trials and kidney disease awareness and prevention programs in the general population, which may ultimately decrease the burden of ESKD.

## Introduction

Epidemiological studies indicate that the metabolic syndrome and its components (elevated blood pressure, elevated triglycerides, low HDL cholesterol, impaired fasting blood glucose and central obesity) are associated with increased cardiovascular morbidity and all-cause mortality in the general population^1^. Furthermore, the metabolic syndrome is linked to the development and progression of chronic kidney disease (CKD)^2–4^. A meta-analysis of 31 studies clearly showed an association between the metabolic syndrome and cardiovascular disease, as well as kidney disease^5^. CKD itself is associated with a large burden of morbidity and mortality^6,7^, exceeding the mortality risk of the general population by 10- to 20-fold^8^.

Approximately one in 40 middle-aged men and one in 60 women will develop end-stage kidney disease (ESKD) during their lifetime^9^. There is some evidence to suggest that the presence of the metabolic syndrome and its components is associated with an increased risk for ESKD rather than an increased risk for death^10^. However, estimating the ESKD risk requires a careful approach that considers the competing event death before ESKD. As shown by Turin *et al*. the lifetime risk for ESKD in the general population is significantly attenuated with the competing risk death before ESKD (relative risk reduction in men by 36%, in women by 23%). In contrast, adjusting for the competing risk of death does not affect ESKD risk in people with impaired kidney function^9^. Evaluation of the influence of known risk factors on the long-term risk for these two competing events might provide new insights to strengthen risk reduction efforts in the general population to prevent ESKD-associated burden of morbidity and treatment costs.

Such studies applying cause-specific risk models in the general population are limited. Previous studies were performed in populations at high risk for ESKD, with either preexisting CKD^10^, hypertension^11^, diabetes mellitus^12^ or cardiovascular disease^13^, but not in a large general population-based cohort without a priori predefined increased risk for kidney disease. Moreover, these studies included older patients^10,11,14^ and used either only clinical^10^ or administrative data sets^11,12,15^.

The objectives of the present prospective, longitudinal study were to investigate the possible influence of blood pressure and metabolic factors on the long-term risks for ESKD and death before ESKD in a large general population based cohort with long follow-up. In addition, these associations were explored in three ESKD high-risk subgroups encompassing participants with prevalent obesity, diabetes mellitus and hypertension.

## Results

### Study cohort characteristics

The analyzed cohort included 177,255 participants (53.8% women) with a mean age at baseline of 42.5 (SD 15.4) years and 2,829,500 person-years of follow-up taking part in the Vorarlberg Health Monitoring and Prevention Program (VHM&PP) (Table 1). By merging the VHM&PP data and the OEDTR data, we identified patients with ESKD requiring dialysis or kidney transplantation, who had also taken part in the VHM&PP program. During a mean follow-up time of 16.0 (SD 5.3) years 358 patients reached ESKD (135 women and 223 men, 0.2%, 13.4 per 100,000 person years), and 19,512 participants died (11%, 730 per 100,000 person years). Mean age at the start of renal replacement therapy was 63.7 (SD 12.7) years and mean time from baseline until ESKD was 10.5 (SD 5.5) years. Overall, 11.4% of the participants were obese, 34.2% hypertensive and 30.0% smokers. Of the participants reaching ESKD 23.2% were obese, 76.4% hypertensive and 39.7% smokers.

**Table 1:** Baseline characteristics of the study population.

	Total		No ESKD		ESKD	
	n	Mean (SD)	n	Mean (SD)	n	Mean (SD)
Age at baseline [years]	177,255	42.5 (15.4)	176,897	42.5 (15.4)	358	53.3 (12.3)
Follow-up [years]	177,255	16.0 (5.3)	176,897	16.0 (5.3)	358	17.9 (4.1)
Age at ESKD [years]	358	63.7 (12.7)	—	—	358	63.7 (12.7)
Time baseline until ESKD [years]	358	10.5 (5.5)	—	—	358	10.5 (5.5)
**Sex**	**n**	**%**	**n**	**%**	**n**	**%**
Female	95,417	53.8	95,282	53.9	135	37.7
Male	81,838	46.2	81,615	46.1	223	62.3
**Mortality**	**n**	**%**	**n**	**%**	**n**	**%**
Death by any cause	19,512	11.0	19,336	10.9	182	50.8
Survived	157,743	89.0	157,561	89.1	176	49.2
**Smoking status**	**n**	**%**	**n**	**%**	**n**	**%**
Non-smoker	124,152	70.0	123,936	70.1	216	60.3
Smoker	53,103	30.0	52,961	29.9	142	39.7
**Obesity**	**n**	**%**	**n**	**%**	**n**	**%**
No	157,023	89.6	156,748	88.6	275	76.8
Yes	20,232	11.4	20,149	11.4	83	23.2
**Hypertension**	**n**	**%**	**n**	**%**	**n**	**%**
No	111,151	62.8	111,067	62.8	84	23.6
Yes	65,965	34.2	65,693	37.2	272	76.4

Abbreviations: ESKD, end-stage kidney disease; SD, standard deviation.

### Association between risk factors and the competing risks ESKD and death before ESKD in the entire cohort

As shown in Table 2 for the entire study population, the fully adjusted Cox regression model (HR, 95% confidence interval) using continuous exposure variable measures revealed stronger associations with ESKD than with death before ESKD for increasing systolic blood pressure [per 5 mmHg HR 1.11 (1.07–1.14) versus 1.03 (1.02–1.03)], fasting blood glucose levels [per mmol/L HR 1.20 (1.16–1.24) versus 1.07 (1.06–1.07)], triglyceride levels [per log mmol/L HR 1.58 (1.29–1.94) versus 1.10 (1.07–1.14)] and total cholesterol levels [per log mmol/L HR 1.16 (1.07–1.26) versus 0.95 (0.94–0.96)]. Increased GGT levels were not associated with ESKD risk, but with increased risk for death [per log U/L HR 1.36 (1.32–1.38)].

**Table 2:** Independent association between risk factors (continuous variables) and ESKD and death before ESKD in the entire cohort.

Risk Factor	ESKD	Death before ESKD
	HR (95% CI)	HR (95% CI)
Cases/n	353/176,190	19,303/ 176,190
Smoking status [smokers vs. non-smokers]	**1.48 (1.18–1.85)**	**1.43 (1.39–1.48)**
BMI [kg/m^2^]	1.01 (0.98–1.04)	0.99 (0.99–0.99)
Fasting blood glucose [mmol/L]	**1.20 (1.16–1.24)**	**1.07 (1.06–1.07)**
Systolic blood pressure [5 mmHg]	**1.11 (1.07–1.14)**	**1.03 (1.02–1.03)**
Diastolic blood pressure [5 mmHg]	**1.08 (1.02–1.14)**	1.00 (0.99–1.01)
Log_triglycerides [mmol/L]	**1.58 (1.29–1.94)**	**1.10 (1.07–1.14)**
Total cholesterol [mmol/L]	**1.16 (1.07–1.26)**	**0.95 (0.94–0.96)**
Log_GGT [U/L]	1.04 (0.89–1.22)	**1.35 (1.32–1.38)**

All cause-specific models are fully adjusted for sex and age at baseline. The HR corresponds to a unit change in the quantity in square brackets.

Abbreviations: ESKD, end-stage kidney disease; CI, confidence interval, GGT; γ-glutamyltransferase.

Conversation factor: To convert glucose from mmol/L to mg/dL, multiply by 18.01; triglycerides from mmol/L to mg/dL, multiply by 88.57; cholesterol from mmol/L to mg/dL, multiply by 38.67.

**Figure 1:**
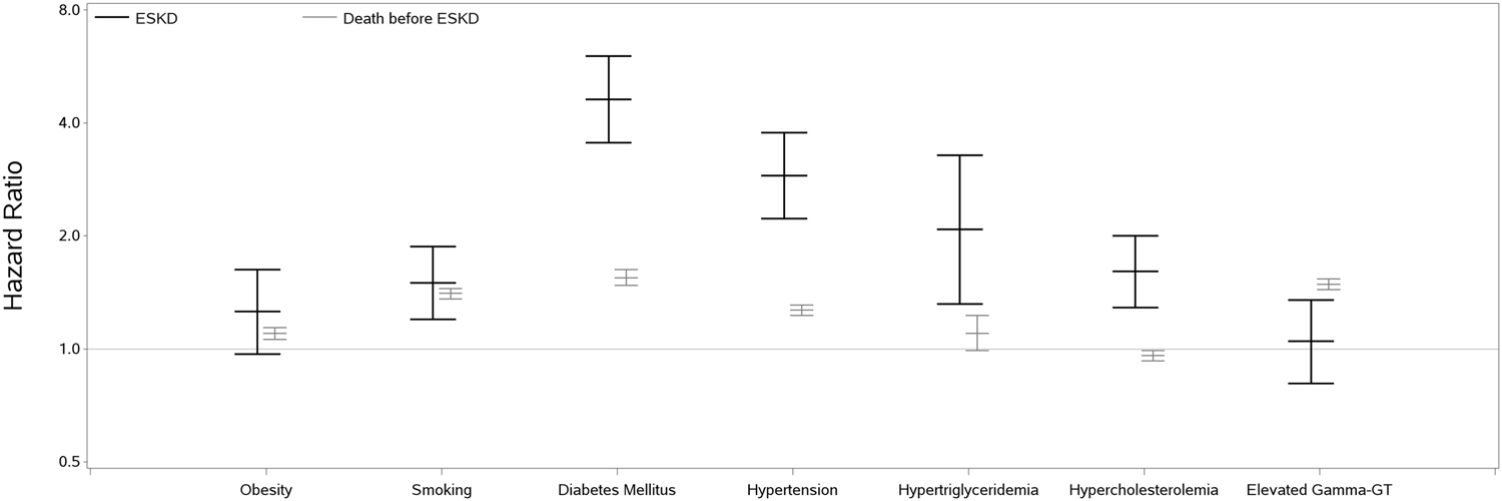
Cause-specific hazard ratios for ESKD and Death before ESKD Hazard ratios are taken from Table 3. All cause-specific models are fully adjusted. Obesity: BMI ≥ 30 kg/m^2^; diabetes mellitus: fasting blood glucose ≥6.9 mmol/L; hypertension: systolic blood pressure ≥140 mmHg, diastolic blood pressure ≥90 mmHg or being on antihypertensive therapy; hypertriglyceridemia: triglycerides ≥2.3 mmol/L; hypercholesterolemia: total cholesterol ≥6.2 mmol/L; elevated gamma-GT: for men ≥61 U/L, for women ≥36 U/L.

Models with exposure variables categorized according to clinically relevant cut-offs showed stronger associations with ESKD than with death before ESKD for diabetes mellitus, hypertension, hypertriglyceridemia and hypercholesterolemia (Fig. 1 and Table 3). The relative risk (HR, 95% confidence interval) for ESKD was highest for participants with diabetes mellitus (HR 4.62, 3.54–6.03), followed by hypertension (HR 2.89, 2.22–3.77), hypertriglyceridemia (HR 2.08, 1.32–3.28) and hypercholesterolemia (HR 1.61, 1.29–2.00).

**Table 3:** Independent association between risk factors (categorical variables) and ESKD and death before ESKD in the entire cohort.

Risk Factor	Categories	ESKD	Death before ESKD
		HR (95% CI)	HR (95% CI)
Cases / n		353/176,190	21,496/176,190
Obesity	No	1.00	1.00
	Yes	1.26 (0.97–1.63)	**1.10 (1.06–1.14)**
Smoking status	Non-smoker	1.00	1.00
	Smoker	**1.50 (1.10–1.71)**	**1.41 (1.36–1.45)**
Diabetes mellitus	No	1.00	1.00
	Yes	**4.62 (3.54–6.03)**	**1.55 (1.48–1.63)**
Hypertension	No	1.00	1.00
	Yes	**2.89 (2.22–3.77)**	**1.27 (1.23–1.31)**
Hypertriglyceridemia	No	1.00	1.00
	Yes	**2.08 (1.32–3.28)**	1.10 (0.99–1.23)
Hypercholesterolemia	No	1.00	1.00
	Yes	**1.61 (1.29–2.00)**	**0.96 (0.93–0.99)**
GGT	Normal	1.00	1.00
	Elevated	1.05 (0.81–1.35)	**1.49 (1.44–1.54)**
	(Men: ≥61 U/L		
	Women: ≥36 U/L)		

Categorical data were used. All cause-specific models are fully adjusted. Obesity: BMI ≥30 kg/m^2^; diabetes mellitus: fasting blood glucose ≥6.9 mmol/L; hypertension: systolic blood pressure ≥140 mmHg, diastolic blood pressure ≥90 mmHg; hypertriglyceridemia: triglycerides ≥2.3 mmol/L; hypercholesterolemia: total cholesterol ≥6.2 mmol/L.

Abbreviations: ESKD, end-stage kidney disease; CI, confidence interval; GGT, γ-glutamyltransferase.

Conversation factor: To convert glucose from mmol/L to mg/dL, multiply by 18.01; triglycerides from mmol/L to mg/dL, multiply by 88.57; cholesterol from mmol/L to mg/dL, multiply by 38.67.

### Association between risk factors and the competing risks ESKD and death before ESKD in selected high-risk populations

Table 4 summarizes cause-specific relative risks in the three selected high-risk populations of obese, hypertensive and diabetic participants using cause-specific Cox regression risk models. Among obese participants, stronger associations with ESKD than with death before ESKD were found for fasting blood glucose (per mmol/L HR 1.27, 1.20–1.34), diastolic blood pressure (per 5 mmHg HR 1.18, 1.05–1.32) and total cholesterol levels (per mmol/L HR 1.33, 1.16–1.51). In hypertensive participants, a higher relative risk for ESKD was found in participants with increasing blood levels of fasting glucose (HR 1.19, 1.14–1.23), triglycerides (HR 1.63, 1.30–2.05) and total cholesterol (HR 1.18, 1.08–1.29), increasing systolic (HR 1.11, 1.07–1.15) and diastolic (HR 1.06, 1.00–1.13) blood pressure and smokers (HR 1.44, 1.11–1.87). Among diabetic participants a higher relative risk for ESKD than for death was detected for increasing fasting blood glucose levels (HR 1.16, 1.10–1.23), systolic blood pressure (HR 1.09, 1.03–1.17) and total cholesterol levels (HR 1.19, 1.05–1.36). In all three high-risk groups, increasing GGT levels revealed a stronger association with death before ESKD than with ESKD (obesity HR 1.36, 1.29–1.43; hypertension HR 1.33, 1.30–1.36; and diabetes mellitus HR 1.25, 1.18–1.32).

**Table 4:** Cause-specific risk models for ESKD and death before ESKD in high-risk participants.

	Obese		Hypertensive		Diabetic	
	ESKD	Death before ESKD	ESKD	Death before ESKD	ESKD	Death before ESKD
Risk Factor	**HR**	**HR**	**HR**	**HR**	**HR**	**HR**
	**(95% CI)**	**(95% CI)**	**(95% CI)**	**(95% CI)**	**(95% CI)**	**(95% CI)**
Cases / n	83/20,137	3,347/20,137	271/65,555	13,532/65,555	78/6,219	2,028/6,168
Smoking status [smokers vs. non-smokers]	1.27	**1.25**	**1.44**	**1.36**	**1.71**	**1.25**
	(0.79–2.03)	**(1.15–1.36)**	**(1.11–1.87)**	**(1.34–1.45)**	**(1.05–2.78)**	**(1.13–1.39)**
BMI [kg/m^2^]	1.04	**1.04**	0.99	1.00	1.02	1.00
	(0.98–1.10)	**(1.03–1.05)**	(0.96–1.02)	(0.99–1.00)	(0.97–1.06)	(0.99–1.01)
Fasting blood glucose [mmol/L]	**1.27**	**1.05**	**1.19**	**1.06**	**1.16**	**1.05**
	**(1.20–1.34)**	**(1.04–1.07)**	**(1.14–1.23)**	**(1.06–1.07)**	**(1.10–1.23)**	**(1.03–1.06)**
Systolic blood pressure [5 mmHg]	1.03	**1.02**	**1.11**	**1.02**	**1.09**	**1.01**
	(0.96–1.10)	**(1.01–1.03)**	**(1.07–1.15)**	**(1.02–1.03)**	**(1.03–1.17)**	**(1.00–1.02)**
Diastolic blood pressure [5 mmHg]	**1.18**	0.99	**1.06**	1.00	1.06	1.01
	**(1.05–1.32)**	(0.98–1.01)	**(1.00–1.13)**	(0.99–1.01)	(0.95–1.19)	(0.99–1.03)
Log_triglycerides [mmol/L]	1.40	1.07	**1.63**	**1.09**	1.29	**1.09**
	(0.93–2.11)	(0.99–1.15)	**(1.30–2.05)**	**(1.05–1.13)**	(0.87–1.92)	**(1.00–1.19)**
Total cholesterol [mmol/L]	**1.33**	1.00	**1.18**	0.96	**1.19**	0.99
	**(1.16–1.51)**	(0.97–1.03)	**(1.08–1.29)**	(0.95–0.98)	**(1.05–1.36)**	(0.95–1.02)
Log_GGT [U/L]	0.74	**1.36**	0.94	**1.33**	1.00	**1.25**
	(0.53–1.05)	**(1.29–1.43)**	(0.79–1.13)	**(1.30–1.36)**	(0.74–1.36)	**(1.18–1.32)**

Continuous data were used. All models are fully adjusted. The HR corresponds to a unit change in the quantity in square brackets.

Abbreviations: ESKD, end-stage kidney disease; GGT, γ-glutamyltransferase.

## Discussion

The main findings of our large population-based cohort study using cause-specific competing risk analyses are that various metabolic risk factors and blood pressure are associated with a higher relative risk for ESKD than for death before ESKD. When investigating the effects of the individual risk factors, fasting blood glucose, blood pressure, triglycerides, and total cholesterol had a stronger influence on the relative risk for ESKD than on death. Only for GGT was the relative risk for death before ESKD higher than the risk for ESKD. Likewise, when predefined definitions of obesity, diabetes mellitus, hypertension, hypertriglyceridemia and hypercholesterinemia were used as categorical variables, all these clinical indicators had a greater impact on ESKD risk than on risk for death before ESKD.

Our findings, namely that elevated levels of fasting blood glucose, blood pressure and triglycerides were associated with a higher relative risk for ESKD, are in line with previously described results in patients with known CKD Stages 3 and 4^10^. In this study, low HDL cholesterol levels were associated with increased mortality, whereas we found an inverse association for total cholesterol. Contrary to our study, obesity and hypertension were associated with reduced all-cause mortality, which could be explained by the fact that these patients were older (mean age 72.3 years) and already had prevalent CKD.

The association between obesity, ESKD and mortality seems to be complex. The effects of obesity are modified by the absence (so-called metabolic healthy obesity) or presence of other components of the metabolic syndrome. Panwar *et al*. found a decreased risk for ESKD in obese individuals without the metabolic syndrome, but an increased incidence rate in those with the metabolic syndrome^16^. They also showed that high blood pressure, triglycerides and fasting blood glucose increase the risk for ESKD. We confirm and extend these findings in a more than 8-fold larger population-based cohort encompassing younger participants, longer follow-up and additional statistical methods considering the important competing event death before ESKD. In the high-risk group of obese participants in our cohort, fasting blood glucose, diastolic blood pressure and total cholesterol were associated with an increased relative risk for ESKD in cause-specific models. This is consistent with the literature showing that excess weight affects ESKD risk via diabetes mellitus and hypertension^10,17,18^. In line with our observation, although in a population of older age and higher co-morbidity burden, Lohr *et al*.^14^ using a competing risk model found that higher systolic blood pressure was associated with an increased incidence of CKD, but not with increased mortality.

Although no causal inference can be made from an observational study like ours due to its design, one might hypothesize that the higher relative risk for ESKD than for death before ESKD could be explained by the potential pathophysiological mechanisms linking the components of the metabolic syndrome to CKD and ESKD. Insulin resistance leads to inflammation and increased oxidative stress^19^, obesity induces the secretion of pro-inflammatory cytokines such as leptin, interleukin-6 and tumor necrosis factor-alpha by adipose tissue^20^, resulting in glomerulosclerosis due to enhanced expression of intra-renal transforming growth factor-beta^21^. Additionally, obesity leads to glomerular and podocyte hypertrophy and mesangial matrix expansion^22^. Trigylcerides may cause renal damage by promoting pro-inflammatory cytokine production^23^. As our population-based study included a middle-aged cohort, therapeutic interventions may have reduced the cardiovascular mortality associated with these risk factors and allowed survival during the observation period but may have been less successful in preventing progression from CKD to ESKD. In contrast, a variety of other causes, which are not influenced by the risk factors analyzed in our study, for example malignancy, contribute to mortality, therefore increasing absolute risk for death.

In our study, elevated GGT was found to be associated with an increased risk for death from all causes, but not for ESKD. GGT is considered neither a classical cardiovascular metabolic risk factor nor a component of metabolic syndrome^3^. However, increased levels of GGT may be a marker of non-alcoholic fatty liver disease^24^ and be associated with the risk for the metabolic syndrome independently of alcohol intake^25^. In the Framingham Heart Study (mean age 44 years, 52% women, and therefore comparable with our study population) higher GGT levels were prospectively associated with cardiovascular disease and all-cause mortality, even after adjustment for established cardiovascular risk factors and hepatic enzymes^26^. Earlier work in our cohort identified baseline GGT levels as well as a longitudinal increase in GGT as being independently associated with cardiovascular death^27,28^. Our finding of an increased risk for death but not for ESKD with higher GGT levels during the same follow-up might reflect the development of a risk factor cluster that constitutes not only vascular morbidity but also other conditions increasing the risk for death.

Additional subdistribution analyses using the Fine and Gray model^29^ revealed similar results compared to the cause-specific Cox models, supporting a higher relative risk for ESKD than for death before ESKD for fasting blood glucose, blood pressure, triglycerides, and total cholesterol in the whole cohort and also in the three selected high-risk populations of obese, hypertensive and diabetic participants. In this paper, we have chosen to report cause-specific relative risks obtained from Cox models of the cause-specific hazards. Just like a standard Cox model, these relative risks express the effect on the intensity of the event under study, either ESKD or death before ESKD, and it is this relative risk concept that is amenable for our discussion of the results above. Some authors (e.g., Schmoor *et al*.^30^) suggest that one may additionally consider Fine and Gray results as a summary of the cause-specific relative risks, but as stated above, results were numerically comparable and we have therefore chosen not to reproduce them. We also note that numerically comparable results are not uncommon comparing these two models^31^. Another issue is that the interpretation of the results obtained from a Fine and Gray model is currently a subject of debate in the biostatistical literature^32,33^. Also for this reason, we have preferred to report cause-specific hazard ratios.

This is one of the largest population-based studies with long-term follow-up to assess risk for ESKD and death before ESKD and investigate the effects of metabolic factors on that risk. In addition, we applied competing risk regression analyses calculating cause-specific models considering the important competing event death that precludes ESKD as the end-point of interest. Furthermore, we analyzed these risk factors in pre-specified high-risk groups, defined by the presence of diabetes mellitus, hypertension or obesity. The results of these group-specific analyses further support our findings in the general population cohort using clinically relevant cut-points.

Notwithstanding these advantages, several limitations of our study have to be kept in mind. Despite multivariable adjustments for established risk factors, potential residual confounding due to other unmeasured lifestyle or medical factors including physical activity and medical treatment could have influenced the observed associations. In particular, information on estimated or measured glomerular filtration rate und albuminuria as markers of renal function at baseline was lacking, because these variables were not included in the VHM&PP (starting in 1988). The ESKD incidence rates during the study period were 14.5 per 100,000 persons per year for the inhabitants of Vorarlberg in general and 13.4 per 100,000 persons per year for the VHM&PP cohort. This slightly lower incidence rate in the VHM&PP cohort is likely due to the fact that healthier persons tend to participate in prevention programs. This should be considered when generalizing the results. The VHM&PP program started enrollment nearly 30 years ago. Metabolic risk management has improved during this time, affecting the relative risk for ESKD and death. However, precisely due to the remote start of the program this study stands out for its long follow-up of 16 years.

The influence of blood pressure and the metabolic factors diabetes mellitus, hypertriglyceridemia and hypercholesterolemia on the relative risk for ESKD found in our study may contribute to stimulate kidney function screening and awareness programs in individuals at risk. Targeting these factors might allow better dialysis-free survival. As diabetes mellitus, hypertension, hypertriglyceridemia and hypercholesterolemia are associated with a higher relative risk for ESKD, future therapeutic trials aiming at improving these conditions should include ESKD as an outcome of interest.

In summary, components of the metabolic syndrome were associated with a higher relative risk for end-stage kidney disease than for its competing event death in a large general population-based cohort of middle-aged adults. In contrast, elevated GGT levels indicated a higher relative risk for death before ESKD. These risk factors vary in their significance depending on the prevalence of one of the three preexisting cardiovascular high-risk phenotypes diabetes, hypertension or obesity. Such information might improve the design of renal risk factor modification trials and enable risk-adapted chronic kidney disease and end-stage kidney disease awareness and prevention programs in the general population, which may ultimately decrease the burden of chronic kidney disease.

## Methods

### Study Population

Between January 1, 1988 and June 30, 2005 177,255 individuals participated in the Vorarlberg Health Monitoring and Prevention Program (VHM&PP), a general population-based risk factor surveillance program in Vorarlberg, Austria’s westernmost state with approx. 360,000 inhabitants in 2015^34^. Every adult in Vorarlberg above the age of 20 years was invited to participate in the program. The screening examinations were performed by the local general practitioner according to a standard protocol, which was described in detail earlier^35^. The program included a physical examination with measurement of height, body weight, systolic and diastolic blood pressure, total cholesterol, triglycerides, γ-glutamyltransferase (GGT), and fasting blood glucose. Participants’ smoking status was inquired, and participants were categorized into “never smokers” and “ever smokers”. The data collection protocol remained unchanged throughout the years of recruitment, therefore the same factors were available for all 177,255 individuals participating in this study. The choice of risk factors included in this study is based on earlier work describing associations between these factors and ESKD in renal risk populations^10,17^ or between these factors and cardiovascular morbidity and mortality in the general population^27,28,36,37^. All participants gave written informed consent to use their data for scientific purposes.

The VHM&PP cohort was linked with the Austrian Dialysis and Transplant Registry (OEDTR) and the National Mortality Registry. The OEDTR collects data on all patients receiving chronic renal replacement therapy (hemodialysis, peritoneal dialysis, kidney transplantation) in Austria since 1964 and has almost complete follow-up^38^. Ethics approval for this study was obtained from the Ethics Committee of the State of Vorarlberg. All study procedures were performed in accordance with the Declaration of Helsinki and relevant guidelines. Written informed consent was obtained from all VHM&PP participants, and all patients registered in the OEDTR signed a declaration of consent to permit their data to be transferred to the registry.

### Statistical analyses

A prospective cohort study was conducted to describe differences between the risk for ESKD and death before ESKD. Outcomes were defined as ESKD requiring hemo-/peritoneal dialysis or kidney transplantation, and death before ESKD (=death from all causes without prior ESKD). Predictors (exposure variables) were analyzed quantitatively, and were also categorized on the basis of clinically relevant definitions. Thus, BMI was calculated as body weight in kilograms divided by squared height in meters. Obesity was defined according to the WHO definition with BMI ≥30 kg/m^2 ^^39^. Fasting blood glucose levels >6.9 mmol/L were defined as diabetes^40^. Hypertension was defined as systolic blood pressure ≥140 mmHg or diastolic blood pressure ≥90 mmHg or being on antihypertensive therapy^41^. Triglycerides ≥2.3 mmol/L defined hypertriglyceridemia and total cholesterol ≥6.2 mmol/L hypercholesterolemia^42^. For GGT, gender-specific cut-offs according to the local central laboratory were used. For men elevated GGT values were defined as ≥61 U/L, and for women elevated GGT values were defined as ≥36 U/L. Each participant´s first examination was used for baseline exposure data. The follow-up by the Austrian Dialysis and Transplant Registry and the National Mortality Registry is population-based and virtually complete. In the exposure variables only few missing variables occurred, which in relation to the size of the dataset are unlikely to have influenced the results.

Cause-specific risk models were calculated to analyze the association between different risk factors and ESKD and death before ESKD^30^. Participants free of either endpoint on December 31, 2009 (end of follow-up) were administratively censored. Survival time until the initiation of renal replacement therapy or until death served as outcome parameter for the Cox regression models. Due to skewness, triglyceride and GGT values were logarithmized. Separate analyses were done for continuous and categorical exposure variables (with categorization based on clinical definitions as described above). Smoking status (ever *vs* never smoker) was included in both analyses. All models were adjusted for sex, age, smoking status and additionally for all included risk factors. Furthermore, we investigated the association in clinically relevant selected high-risk groups, encompassing participants with prevalent arterial hypertension, diabetes mellitus and obesity. Interaction was investigated by including a product term in the Cox regression equation. All tests were two-sided, and statistical significance was defined as a *P* value ≤ 0.05. All calculations were performed using the statistical analysis software SAS, release 9.3 (SAS Institute, Cary, NC, USA).

### Data availability

The datasets generated during and analyzed during the current study are not publicly available due to the Austrian law which prohibits public availability of health-related personal data but are available from hans.concin@aks.or.at on reasonable request.
